# Correction: Children’s experiences of play in digital spaces: A scoping review

**DOI:** 10.1371/journal.pone.0321031

**Published:** 2025-03-25

**Authors:** Fiona M. Loudoun, Bryan Boyle, Maria Larsson-Lund

The Funding statement for this paper is incorrect. The correct Funding statement is as follows: The project receives funding from the European Union’s Horizon 2020 research and innovation programme under the Marie Skłodowska-Curie grant agreement No 861257.
