# Supplementary figures and images for: Children’s experiences of play in digital spaces: A scoping review
Source: PLoS One. 2022 Aug 9;17(8):e0272630. doi: 10.1371/journal.pone.0272630 (PMC9362941; doi:10.1371/journal.pone.0272630)

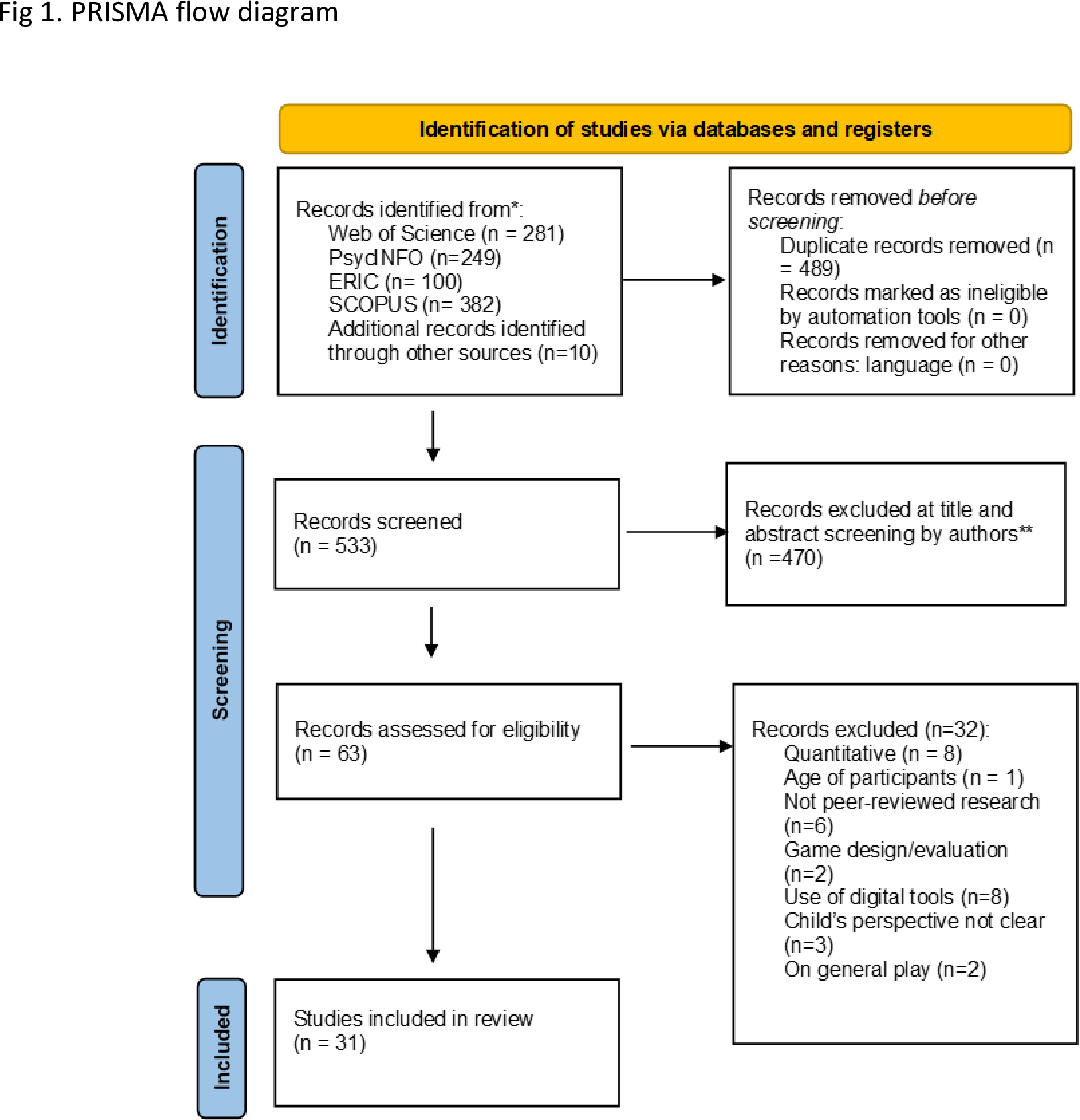

Supplement: S1 Fig — (TIF) [file pone.0272630.s001.tif]
