# Supplementary material for: Children’s experiences of play in digital spaces: A scoping review
Source: PLoS One. 2022 Aug 9;17(8):e0272630. doi: 10.1371/journal.pone.0272630 (PMC9362941; doi:10.1371/journal.pone.0272630)
Supplement: Attachment — Submitted filename: Responsetoreviewers.docx [file pone.0272630.s002.docx]

**Response to Reviewers**

Dear Academic Editor and Reviewers,

Many thanks for taking the time to review the manuscript: PONE-D-22-03218, Children's experiences of play in digital spaces: a scoping review. Please see the responses below responding to each point of your feedback. The response is in italic directly below the comments made by each individual. We hope that the responses are to your satisfaction.

**Academic Editor**

1. Please ensure that your manuscript meets PLOS ONE's style requirements.

***Manuscript has been cross-checked with the formatting guidelines and style requirements.***

2. You have not specified where the minimal data set underlying the results described in your manuscript can be found.

***This is not applicable for the scoping review – the minimal data set has been identified throughout the review. The databases and search terms have been fully described throughout the manuscript.***

3. Should your manuscript be accepted for publication, we will hold it until you provide the relevant accession numbers or DOIs necessary to access your data.

***As highlighted above, this will not be applicable.***

**Reviewer #1**

1. The text refers to both play and free play - if both mean play in the sense of Article 31 UN CRC, then the addition of 'free' is not necessary (this is included in the concept of 'play' in the sense of voluntary and unscripted play); if, according to the authors, there is a difference between the two, then this could be explained.

***Throughout the text, play is referred to as the play in the sense of the UNCRC, therefore, we agree that free is not required as an addition to play. Free has therefore been removed from the text where necessary.***

2. I understand that a clear demarcation is needed, but I find it unfortunate that hybrid forms of play (combination of offline and online play) were not included, since the offline and online daily lives of children are now completely intertwined, and they may no longer make a distinction in their play. In addition, I suspect that one would then also focus the perspective a bit more on the younger children as I expect that especially with them, the form of play is relevant (at least it would be interesting to find out). Perhaps this is something to elaborate on in future research.

***It is particularly evident from the research that children do not differentiate between online and offline play as adults tend to do. There is some research by Kate Cowan and John Potter (***[***https://doi.org/10.1177/2043610620941527***](https://doi.org/10.1177%2F2043610620941527)***), examining how the two intersect. I believe that this will evolve organically as our research progresses.***

3. The article contains a reference to a report by Livingstone and Pothong on playful by design; both scholars recently published an academic paper on the subject that the authors might want to mention in their article: <https://www.tandfonline.com/doi/full/10.1080/1369118X.2022.2046128>

***Thank you for highlighting the academic paper by Livingstone and Pothong. This has been updated accordingly in the manuscript.***

4. I was interested in the 'shift in societal norms' that the authors refer to (see, among others, line 79) but they do not elaborate on it; perhaps this could be explained a bit more.

***This has now been elaborated on to discuss the binaries that play is often positioned within as a result of an adult dominated discussion.***

5. As part of the analysis, the 'personal and ecological relevance' (a.o. line 106) of digital play for children is examined. This is explained later in the article (see line 171), but I would bring the explanation forward. It was not clear to me what the authors meant by it so an earlier explanation would be helpful (and, as far as I am concerned, it could be explained a bit more).

***We have been aware of ensuring that terms are clearly defined given that they may mean different things to different professions. Lines 48; 85-92 define and explain personal and ecological relevance, however, the sentence has been altered to begin: ‘Personal and ecological relevance refers to…’ to ensure it is explicit.***

6. From line 238 onwards, it talks about cheating and meta gaming: can the authors give examples of what is meant by that?

***A definition of metagaming has been included as well as examples of cheating within a virtual world.***

7. Finally, I would read the text again carefully because sometimes sentences did not seem to run quite right.

***A further proofread of the manuscript has been completed.***

**Reviewer #2**

1. The research question itself, especially the second question, is too vague and somewhat convoluted, "The scoping review will examine what evidence is available regarding children’s perspectives of play in digital spaces and how the daily relevance, personal and ecological relevance, and methods are approached within the research articles". Examining children's perspective of play in digital spaces is clear. Does the second question mean to examine methods used by the articles to examine the first question? If so, it shouldn't be "and methods". It may seem an irrelevant distinction, but it makes the reading and purpose a little clearer.

***Thank you for the comment. The order of the words in the second part of the research question provides sequence and cohesion for the findings that follow. However, by adjusting the question to:***

*“The scoping review will examine what evidence is available regarding children’s perspectives of play in digital spaces* ***by exploring how*** *the daily relevance, personal, and ecological relevance and methods are approached within research articles.”* ***May help to make the distinction clearer.***

2. I would also like a clearer definition of "ecological relevance".

***This was also highlighted by Reviewer #1. From lines 85-92 – personal and ecological relevance is defined and described. However, the sentence has been reworded to ensure explicit defining of the terms.***

3. The abstract notes that, "these perspectives are essential for parents, professionals, game designers, and policymakers alike" but there is little discussion of why this is essential. What will parents, for example, learn from their children's perspective of digital play that would impact their parenting?

***Sentence altered to consider that this will inform and contribute to an enhanced understanding of play in digital spaces.***

4. In general, the discussion piece would have benefitted from a clearer delination of the arguments (there was some repetition and unclear links between aspects of the analysis) and the relevance to the lives of children. The reader can get a little lost in the critique of the articles not included children's voices and wish for more analysis of how these digital platforms could/do provide opportunities for child centered play.

***A further proofreading of the document has been completed to ensure a clearer and more succinct analysis.***

5. Proof readings would be helpful i.e. line 484, 442, 512 etc.

***Proof reading of the full document has been completed.***
